# Localized Surface Plasmon Resonance with Five-Branched Gold Nanostars in a Plastic Optical Fiber for Bio-Chemical Sensor Implementation

**DOI:** 10.3390/s131114676

**Published:** 2013-10-29

**Authors:** Nunzio Cennamo, Girolamo D'Agostino, Alice Donà, Giacomo Dacarro, Piersandro Pallavicini, Maria Pesavento, Luigi Zeni

**Affiliations:** 1 Department of Industrial and Information Engineering, Second University of Naples, via Roma 29, 81031 Aversa, Italy; E-Mail: luigi.zeni@unina2.it; 2 Department of Chemistry, University of Pavia, Via Taramelli 12, 27100 Pavia, Italy; E-Mails: girolamo.dagostino@unipv.it (G.D.); alice.dona@gmail.com (A.D.); piersandro.pallavicini@unipv.it (P.P.); maria.pesavento@unipv.it (M.P.); 3 Department of Physics, University of Pavia, Via Bassi 6, 27100 Pavia, Italy; E-Mail: giacomo.dacarro@unipv.it; 4 CNR-IREA via Diocleziano 328, 80124 Naples, Italy

**Keywords:** localized surface plasmon resonance, plastic optical fiber, gold nanostars, chemical sensors

## Abstract

In this paper a refractive index sensor based on localized surface plasmon resonance (LSPR) in a Plastic Optical Fiber (POF), is presented and experimentally tested. LSPR is achieved exploiting five-branched gold nanostars (GNS) obtained using Triton X-100 in a seed-growth synthesis. They have the uncommon feature of three localized surface plasmon resonances. The strongest LSPRs fall in two ranges, one in the 600–900 nm range (LSPR 2) and the other one in the 1,100–1,600 nm range (LSPR 3), both sensible to refractive index changes. Anyway, due to the extremely strong attenuation (>10^2^ dB/m) of the employed POF in the 1,100–1,600 nm range, only LSPR 2 will be exploited for refractive index change measurements, useful for bio-chemical sensing applications, as a proof of principle of the possibility of realizing a compact, low cost and easy-to-use GNS based device.

## 1. Introduction

LSPR, associated with noble metal nanostructures, creates a sharp spectral absorption and scattering peaks as well as strong electromagnetic near-field enhancements. The past decade has witnessed significant improvements in the fabrication of noble metal nanostructures, which has led to advances in several areas of the science and technology of LSPR. Among these, there is the detection of molecular interactions near the nanoparticle surface through shifts in the LSPR spectral peaks [1]. The localized electromagnetic field around the metal surfaces is very sensitive to environmental refractive indexes. Environmental changes, at the interface between media and metals, can be traced by monitoring the changes of metal LSPR characteristics. Sensors based on LSPR in a plastic optical fiber, exploiting gold nanoparticles, present several advantages [2,3]. First, the use of a plastic optical fiber (POF) reduces the cost and the dimension of the device, with the possibility of easy integration of LSPR sensing platform with optoelectronic devices, such as LEDs and photodetectors, and electronic devices for data processing, as well. Second, the multiple reflections of light occurring in the optical fiber allow to excite the sample to a large extend, so the detection sensitivity to the analytes can be improved. Third, the multiple resonances of gold nanostars add flexibility to the sensor's design, making it possible to vary the geometry, the material and the configuration. Therefore, the plastic optical fiber sensor based on LSPR effect in gold nanostars has the benefits of being a compact, low cost and high sensitivity device also allowing remote/online detection. Furthermore, the deposition of a Molecularly Imprinted Polymer (MIP) film on the nanoparticle layer could make the device an extremely selective one. This has been recently demonstrated in the case of a sensor for TNT, based on POF, in which the metal was a uniform gold layer. In that case the resonance wavelength was at around 760 nm [4].

In the present work, a gold nanostars layer instead of a compact gold layer is used and, as a proof of principle, the device was tested against solvents, or solutions with different refractive index, in order to determine its sensitivity to the refractive index changes. It is important to underline that, even if the sensitivity happens to be lower than the one achievable with a compact gold layer when tested just against solvents, a proper exploitation of the tridimensional structure of these nano-objects should allow a better interaction with the specific sites present in the tridimensional structure of the molecularly imprinted polymers, leading to the realization of an extremely efficient sensor.

## 2. Background: LSPR Phenomenon

LSPR is characterized by the resonance peaks. To find the functional form of peaks wavelengths dependence on the dielectric function (ε_1_) of the medium, one can use the analytical, frequency-dependent form for ε_1_ from the Drude model of the electronic structure of metals [1,5]:

(1) $$\varepsilon_{1}=1-\frac{{\omega_{P}}^{2}}{\omega^{2}+\gamma^{2}}$$

where ω_p_ is the plasma frequency and γ is the damping parameter of the bulk metal. The Drude model is a purely classical model of electronic transport in conductors. It describes the collisions between freely moving electrons and a lattice of heavy, stationary ionic cores; it provides a very good approximation of the conductivity of noble metals. For visible and near-infrared frequencies, the inequality γ ≪ ω holds true, so the above equation can be simplified to:

(2) $$\varepsilon_{1}≅1-\frac{{\omega_{P}}^{2}}{\omega^{2}}$$

Using this expression for ε_1_ and setting ε_1_= −2 ε_m_ (the resonance condition), one obtains the following:

(3) $$\omega_{\max}=\frac{\omega_{P}}{\sqrt{2\varepsilon_{m}+1}}$$

where ω_max_ is the LSPR peak frequency. Converting from frequency to wavelength via λ = 2 πc/ω, and then from dielectric constant to index of refraction via ε_m_ = n^2^, the above expression becomes:

(4) $$\lambda_{\max}=\lambda_{p}\sqrt{2{n_{m}}^{2}+1}$$

where λ_max_ is the LSPR peak wavelength and λ_p_ is the wavelength corresponding to the plasma frequency of the bulk metal. Thus, we see that the dependence of LSPR peak wavelength on the refractive index ought to be approximately linear at optical frequencies; this is borne out by experimental results.

The sensitivity (S) of a nanoparticle based sensor can be defined by calculating the shift in resonance wavelength per unit change in refractive index. It is usually reported in nanometers of peak shift per Refractive Index Unit (nm/RIU):

(5) $$S=\frac{\delta \lambda }{\delta n}\left[\frac{nm}{\text{RIU}}\right]$$

The simplest sensing application of LSPR-active particles or nanostructures [6–8] is to detect changes in the bulk refractive index of their environment through shifts in the LSPR peak wavelength. LSPR peaks are typically detected by spectral extinction measurements on a dense film or spectral scattering measurements on single nanoparticles.

Nicely regular five-branched GNS have been recently obtained by some of us, using the non-ionic surfactant Triton X-100 (TX100) in a seed-growth synthesis in water [9]. These GNS exhibit the unusual feature of three localized surface plasmon resonances. While the transversal oscillation of the valence electrons generate a “short” LSPR at ∼530 nm (LSPR 1), the other two plasmon resonances span the near-IR (NIR) interval, entering the short-wavelength IR (SWIR) domain. In particular, the maximum absorption wavelength of these LSPR can be positioned in the 600–900 nm (LSPR 2) and 1,100–1,600 nm (LSPR 3) ranges, respectively, by regulating the reactants concentration, that, in turn, regulates the LWR (length to width ratio) of the branches. All LSPRs are photothermally active, *i.e.*, convert efficiently radiation into heat [9]. These GNS may thus, for instance, be used as tools for nanomedicine exploiting the 700–1,000 nm transparent window of biological matter for through-tissues photothermal treatments against tumors or multi-drug resistant bacterial infections.

## 3. Materials and Methods

### 3.1. Five-Branched Gold Nanostars

#### Preparation of GNS for deposition on POF

Seed solution: In a 20 mL vial, HAuCl_4_ (5 mL, 5 × 10^−4^ M in water) is added to an aqueous solution of TritonX-100 (5 mL, 0.2 M). The mixture is gently hand-shaken and a pale yellow color is obtained. Then, a previously ice-cooled solution of NaBH_4_ (0.6 mL, 0.01 M in water) is added. The mixture is gently hand-shaken and a reddish color appears.

Growth solution (10 mL samples): In a 20 mL vial, AgNO_3_ (180 μL, 0.004 M in water) and HAuCl_4_ (5 mL, 0.001 M in water) are added in this order to an aqueous solution of TritonX-100 (5 mL, 0.2 M). Then, an aqueous solution of ascorbic acid (170 μL, 0.0788 M) is added. The solution, after gentle mixing, becomes colorless. Soon after, the seed solution (12 μL) was added. The solution is gently hand-shaken and a pink color appears and quickly changes to blue and becomes more intense. After 1 h at room temperature PEG2000-SH is added in a concentration of 5 × 10^−5^ M. The mixture is stirred for 1h at room temperature, then the nanoparticles undergo three cycles of ultracentrifugation (13,000 rpm, 11 min)/elimination of the surnatant/redissolution of the pellet in 10 mL of bi-distilled water. These steps are required to eliminate excess PEG2000-SH and TritonX-100. Then, another cycle of ultracentrifugation is performed, the surnatant discarded and the pellet redissolved in 1 mL of bidistilled water to concentrate the particles (10X in respect to the starting colloidal solution). The final concentration is ∼ 0.6 mgAu/mL.

**Figure 1.**
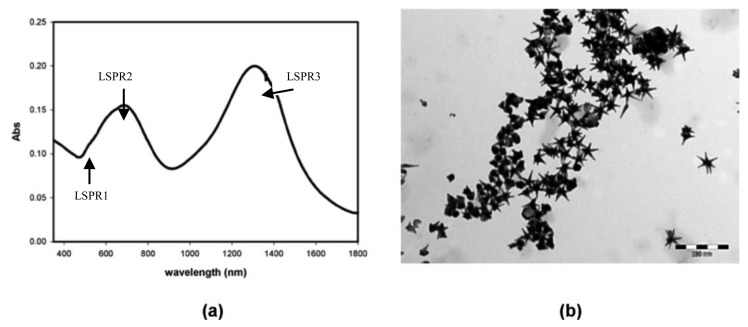
(**a**) UV-Vis-nIR absorption spectrum of the five-branched GNS. (**b**) TEM image of the same sample.

Figure 1a shows the UV-Vis-nIR absorption spectrum of the five-branched GNS used in this work (sample is diluted 1:10 with bidistilled water and the spectrum is registered on a Cary6000i UV-Vis-nIR spectrophotometer equipped with a 1 mm glass cuvette) and Figure 1b shows a TEM image of the same sample (acquired on a Jeol JEM-1200 EX II 140 instrument).

### 3.2. POF Sensor System

#### 3.2.1. Preparation of POF

The optical sensor was realized removing the cladding of a plastic optical fiber along half a circumference. The plastic optical fiber has a PMMA core of 980 μm and a fluorinated cladding of 20 μm, so it is multimode in the considered spectral range (700–740 nm) with an average attenuation of about 0.5 dB/m. The sensing region is about 10 mm in length. The POF was embedded in a resin block, with the purpose of easing the polishing process. The polishing process was carried out with a 5 μm polishing paper in order to remove the cladding and part of the core. After 20 complete strokes following a “8-shaped” pattern in order to completely expose the core, a 1 μm polishing paper was used for another 20 complete strokes with the same pattern [10]. Finally, the gold nanostars layer is deposited.

#### 3.2.2. Deposition of Gold Nanostars on POF

**Figure 2.**
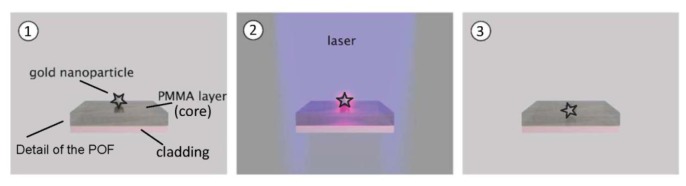
Scheme of immobilization steps: before (1), during (2), and after (3) laser irradiation.

**Figure 3.**
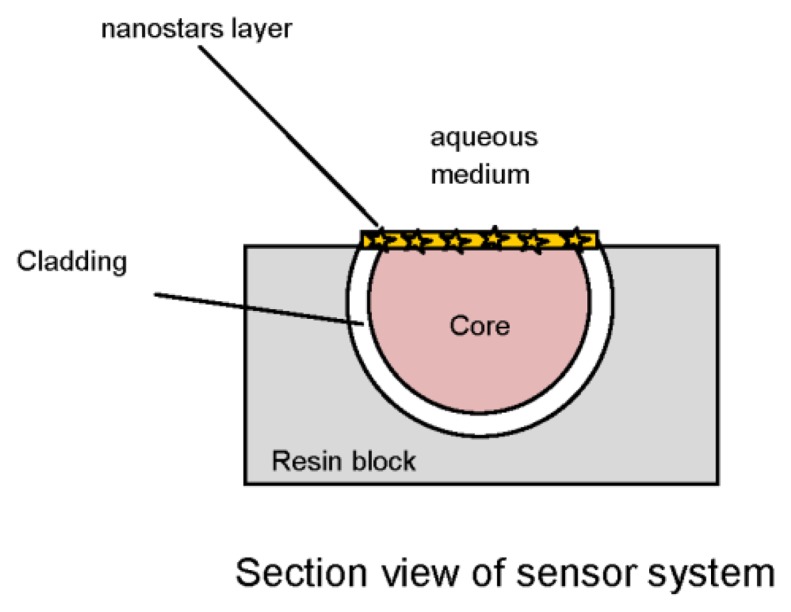
Optical sensor system based on LSPR in POF.

The gold nanostars solution (5 μL, 0.6 mgAu/mL) is dropped on the plastic optical fiber. The solution is dried in vacuum for 30 min. The entire procedure is repeated a second time. The heat-releasing feature of GNS, when laser irradiated at the resonance wavelengths, is exploited to immobilize them on the POF core. Figure 2 illustrates the immobilization steps [11]. In particular, a compact CW laser with a wavelength of 532 nm and an output power of 50 mW was used. Figure 3 shows the detail of optical sensor system.

### 3.3. Experimental Setup

**Figure 4.**
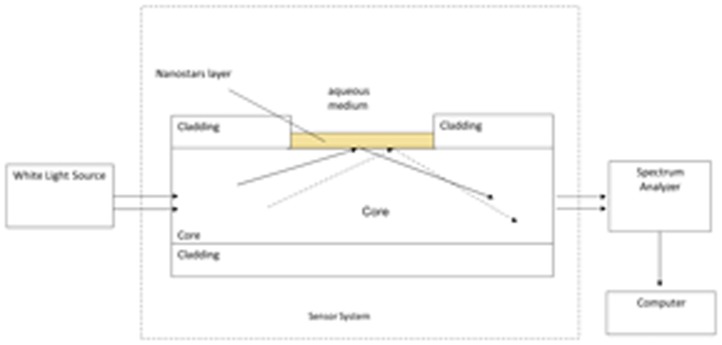
Setup to measure the transmitted light spectrum.

The experimental setup was arranged to measure the transmitted light spectrum and was characterized by a halogen lamp to illuminate the optical sensor system, and a spectrum analyzer, as shown in Figure 4. The employed halogen lamp (Model No. HL-2000-LL, manufactured by Ocean Optics) exhibits a wavelength emission range from 360 nm to 1,700 nm, while the spectrum analyzer detection range was from 350 nm to 1,100 nm. An Ocean Optics “USB2000+VIS-NIR-ES” spectrometer has been employed. The spectrometer was finally connected to a computer. The LSPR curves along with data values were displayed online on the computer screen and saved with the help of advanced software provided by Ocean Optics.

In this case, the resolution (Δn) of the LSPR-based optical sensor can be defined as the minimum amount of change in refractive index detectable by the sensor. This parameter definitely depends on the spectral resolution (δλ_DR_) of the spectrometer used to measure the resonance wavelength. In this configuration, the spectral resolution (δλ_DR_) of the spectrometer was 1.5 nm (FWHM). Therefore, if there is a shift of δλ_res_ in resonance wavelength corresponding to a refractive index change of δn, then resolution can be defined as:

(6) $$\Delta n=\frac{\delta n}{\delta \lambda_{\text{res}}}\delta \lambda_{DR}$$

### 3.4. Solubilization of GNS in Different Solvents

A sample of GNS (40 mL) is prepared following the procedure reported in Section 3.1. After pegylation, the sample undergoes a cycle of ultracentrifugation/elimination of the surnatant/redissolution of the pellet in water. Then, the sample is divided into eight portions (5 mL each) and these are ultracentrifugated (13,000 rpm, 11 min), the surnatant is discarded and the pellets are dried under vacuum. Each portion is redissolved in 5 mL of the following solvents: water, ethanol, *n*-butanol, chloroform, ethyl acetate, toluene, acetonitrile, N,N-dimethylformamide. The absorption spectrum of each portion is registered on the UV-Vis-nIR spectrophotometer Cary6000i equipped with a glass cuvette with an optical path of 1 mm.

## 4. Results and Discussion

### 4.1. GNS Deposited on POF

In the series of performed experiments with the POF sensor, water-glycerin solutions were used to achieve an aqueous medium with variable refractive index. The choice of water-glycerin solution (maximum refractive index 1.474, 100% glycerin) stems from the fact that our device works for refractive indexes around 1.350, and water-glycerin mixtures are easy to prepare and their refractive indexes can be easily measured, by using an Abbe refractometer, allowing one to obtain solutions with small index steps in the working range of the sensor. The measurements were carried out by dropping 50 μL of water-glycerin solutions over the sensing head and letting the drops expand over the whole sensing area. In this case, among the maximum absorption ranges, we focus on LSPR 2 because it falls just in the spectral interval that can be investigated with the POF sensor.

**Figure 5.**
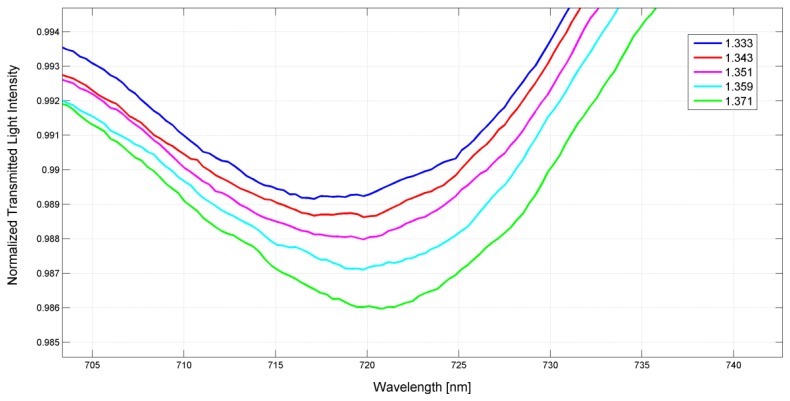
Experimentally obtained LSPR transmission spectra, normalized to the air spectrum, for different refractive index of the aqueous medium.

Figure 5 presents the experimentally obtained LSPR 2 transmission spectra, normalized to the spectrum with air as the surrounding medium (reference spectrum), for different water-glycerin solutions with refractive index ranging from 1.333 to 1.371. It is seen that an increase of the refractive index moves toward higher values the resonance wavelength.

**Figure 6.**
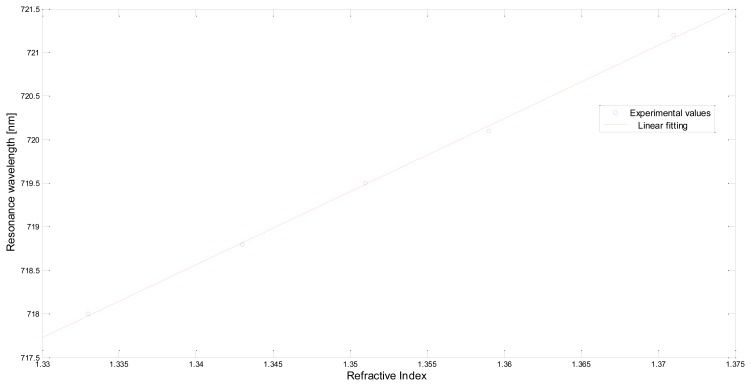
Plasmon resonance wavelength as a function of the refractive index (for LSPR 2), when the gold nanostars are immobilized on the PMMA.

Figure 6 shows the resonance wavelength versus the refractive index, for five refractive index values (circles), along with the linear fitting to the experimental data. The device sensitivity can be measured as the shift of the resonance wavelength (nm) per unit change in refractive index (nm/RIU). As the data show a good linearity, the Pearson's correlation coefficient being equal to 0.999, the sensitivity is conveniently represented by the angular coefficient of the linear fitting shown in Figure 6, *i.e.*, 84 [nm/RIU].

### 4.2. GNS Suspended in Different Solvents

**Figure 7.**
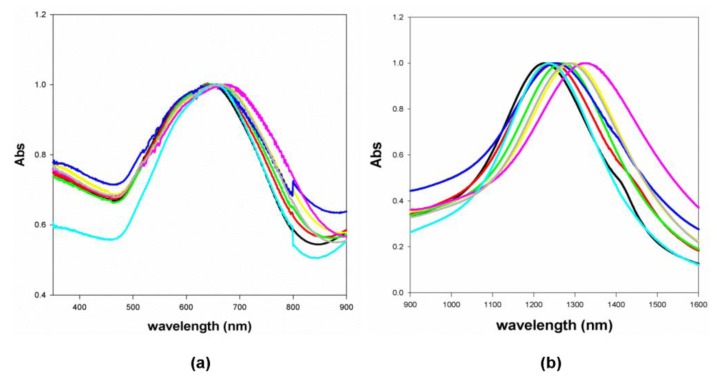
Normalized absorption spectra (a: LSPR2, b: LSPR3) of GNS suspended in different solvents. Black: water (n = 1.3300); light blue: acetonitrile (n = 1.3442); red: ethanol (n = 1.3611); blue: ethyl acetate (n = 1.3723); green: *n*-butanol (n = 1.3990); grey: N,N-dimethylformamide (n = 1.4300); yellow: chloroform (n = 1.4476); magenta: toluene (n = 1.4961).

For comparison purposes, Figure 7 shows the normalized absorption spectra of GNS suspended in different solvents with different refractive indexes (see Section 3.4). In this case, instead of water-glycerin mixtures, liquids with different refractive index have been used to allow a more complete characterization of the resonances, also well beyond the working range of the POF sensor. Surface modification of gold nanostars by means of PEG2000-SH allows preventing the aggregation of the nanoparticles, thanks to the amphiphilic characteristic of the polymer (it is soluble both in water and organic solvents).

**Figure 8.**
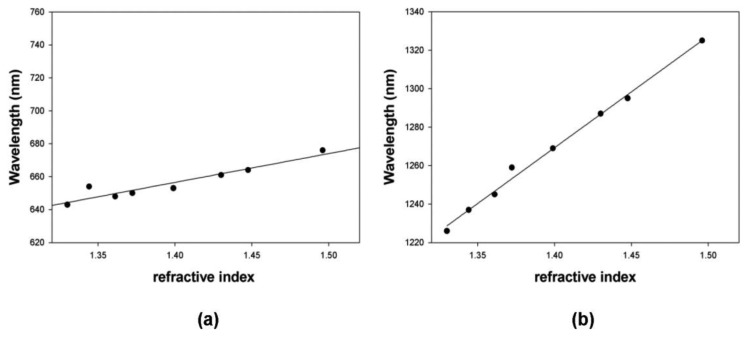
Plasmon resonance wavelength as a function of the refractive index (a: LSPR 2, b: LSPR 3), when the gold nanostars are suspended in different solvents: water (n = 1.3300); acetonitrile (n = 1.3442); ethanol (n = 1.3611); ethyl acetate (n = 1.3723); *n*-butanol (n = 1.3990); N,N-dimethylformamide (n = 1.4300); chloroform (n = 1.4476); toluene (n = 1.4961).

It has to be noticed that both LSPR 2 and LSPR 3 show a dependence of the peak position with refractive index of the medium. The linear fitting to the experimental data, for the two plasmon resonances, are shown in Figure 8 and the sensitivities are 175 [nm/RIU] for LSPR 2 and 580 [nm/RIU] for LSPR 3, respectively. It is important to stress that LSPR 2 sensitivity, shown in Figure 8a, is larger than the POF sensor one, because in this case the entire surface of the gold nanostars is in contact with the liquid.

### 4.3. Discussion

The measured sensitivity of POF sensor system is about half the sensitivity exhibited by GNS suspended in a aqueous medium (see Figures 6 and 8a). This is due to the fact that, when the GNS are immobilized on the POF core, the refractive index “seen” by the GNS can be regarded as the average of the refractive index of PMMA (POF core) and the refractive index of the aqueous medium. By employing a first-order approach, it can be stated that half of gold nanostars surface is embedded in PMMA while the other half is in contact with the aqueous medium. Therefore, the sensitivity is about 50% of that achieved with the GNS suspended in the aqueous medium. Moreover, the resonance wavelengths, when the GNS are immobilized on PMMA, are red-shifted with respect to the GNS suspended in the aqueous medium (see Figures 1 and 5), as expected for a higher index medium.

It is important to underline two aspects relative to the sensitivity of the proposed device: first, the reduced sensitivity, with respect to the suspended nanostars, is a drawback of the proposed POF integrated device that is somehow compensated by the possibility to realize a compact, highly selective and easy-to-use sensor; second, the reduced sensitivity with respect to similar POF devices, where a gold film is employed, can be overcome by using a 3-D distribution of nanostars embedded in a selective layer, such as an MIP layer, so allowing the simultaneous interrogation of many sites, eventually leading to an even higher sensitivity.

## 5. Conclusions and Future Trends

A sensor based on LSPR in a plastic optical fiber, useful for bio-chemical sensing applications, has been realized and experimentally tested. The proposed device is based on the excitation of localized surface plasmons at the interface between under test medium and a nanostars layer deposited on the fiber core. The sensing device has been characterized by exploiting a halogen lamp to illuminate the optical fiber and observing the transmitted spectra, normalized to the spectrum transmitted when the outer medium is air. The experimental results indicate that the configuration exhibits good performance in terms of sensitivity. Furthermore, the proposed sensing head, being low cost and relatively easy to realize, may be very attractive for bio-chemical sensors implementation [4,12,13]. Work is in progress to exploit an MIP layer combined with GNS in order to achieve highly selective detections of specific analytes and higher sensitivity.
